# Blood DCs activated with R848 and poly(I:C) induce antigen-specific immune responses against viral and tumor-associated antigens

**DOI:** 10.1007/s00262-021-03109-w

**Published:** 2021-11-25

**Authors:** Gerulf Hänel, Caroline Angerer, Katja Petry, Felix S. Lichtenegger, Marion Subklewe

**Affiliations:** 1 Department of Medicine III, University Hospital, LMU Munich, Marchioninistr. 15, 81377 Munich, Germany; 2 Laboratory for Translational Cancer Immunology, Gene Center, LMU Munich, Munich, Germany; 3 Miltenyi Biotec B.V. & Co. KG, Bergisch Gladbach, Germany; 4 German Cancer Consortium (DKTK) and German Cancer Research Center (DKFZ), Heidelberg, Germany; 5 Present Address: Roche Innovation Center Munich, Penzberg, Germany

**Keywords:** Immunotherapy, Blood dendritic cells, Toll-like receptors, Plasmacytoid dendritic cells, Conventional dendritic cells

## Abstract

Monocyte-derived Dendritic cells (DCs) have successfully been employed to induce immune responses against tumor-associated antigens in patients with various cancer entities. However, objective clinical responses have only been achieved in a minority of patients. Additionally, generation of GMP-compliant DCs requires time- and labor-intensive cell differentiation. In contrast, Blood DCs (BDCs) require only minimal ex vivo handling, as differentiation occurs in vivo resulting in potentially better functional capacities and survival. We aimed to identify a protocol for optimal in vitro activation of BDCs including the three subsets pDCs, cDC1s, and cDC2s. We evaluated several TLR ligand combinations and demonstrated that polyinosinic:polycytidylic acid [poly(I:C)] and R848, ligands for TLR3 and TLR7/8, respectively, constituted the optimal combination for inducing a positive co-stimulatory profile in all BDC subsets. In addition, TLR3 and TLR7/8 activation led to high secretion of IFN-α and IL-12p70. Simultaneous as opposed to separate tailored activation of pDCs and cDCs increased immunostimulatory capacities, suggesting that BDC subsets engage in synergistic cross-talk during activation. Stimulation of BDCs with this protocol resulted in enhanced migration, high NK-cell activation, and potent antigen-specific T-cell induction.

We conclude that simultaneous activation of all BDC subsets with a combination of R848 + poly(I:C) generates highly immunostimulatory DCs. These results support further investigation and clinical testing, as standalone or in conjunction with other immunotherapeutic strategies including adoptive T-cell transfer and checkpoint inhibition.

**Supplementary Information:**

The online version contains supplementary material available at 10.1007/s00262-021-03109-w.

## Introduction

Generating, enhancing, and maintaining tumor-specific immune responses is a major challenge in the search for cures for cancer. Dendritic cells (DCs) orchestrate innate and adaptive immunity, thereby inducing tailored, strong, and durable immune responses. They have therefore been extensively evaluated as an immunotherapeutic tool, including various in vivo targeting approaches [1, 2]. However, most pre-clinical and clinical studies have focused on ex vivo DC activation and antigen-loading approaches [3, 4].

Numerous clinical trials have evaluated monocyte-derived DCs (moDCs) against different cancer entities, including multiple myeloma and Acute myeloid leukemia (AML). Their overall safety and induction of tumor-specific immune responses have been demonstrated [4–8]. However, objective clinical responses have only been obtained for a minority of patients [9, 10]. In addition, the generation of moDCs is labor- and time-consuming as the differentiation of monocytes into DCs takes several days. Furthermore, transcriptional comparisons of moDCs and naturally occurring DC subsets indicate great functional differences [11].

Therefore, other sources of DCs have recently been evaluated. Primary blood DCs (BDCs) are hypothesized to be ideal candidates for inducing anticancer immune responses since they differentiate in vivo and require only brief ex vivo handling, resulting potentially in better preservation of functional capacities and longer in vivo survival [12]. At least three BDC populations can be distinguished: CD11c^+^ CD141^+^ CLEC9A^+^ conventional DCs (cDC1s), CD11c^+^ CD1c^+^ cDC2s, and CD11c^−^ CD303^+^ CD123^+^ plasmacytoid DCs (pDCs) [13].

pDCs are specialized to recognize viral infections via toll-like receptor (TLR) 7 or TLR9, which results in strong production of type I interferons (IFNs). These cytokines modulate adaptive and innate immunity by inducing, for example, NK-cell activation, B-cell differentiation, and Th1 polarization [14]. In contrast, cDC2s express TLRs that recognize lipopolysaccharides (TLR4), flagellin (TLR5), and the lipoproteins of bacteria and fungi (TLR2 and TLR6). They also express TLR8 and TLR9 that are activated by single-stranded RNA and DNA, respectively. Thus, different CD4^+^ T helper subsets and CD8^+^ T cells are induced depending on the circumstances [15]. cDC1s represent a rare BDC subset that expresses, among others, TLR3 that is activated by viral double-stranded RNAs leading to secretion of interleukin (IL) 12 [16]. They also express TLR8, which is closely related to TLR7, and the endocytic receptor CLEC9A that promotes antigen cross-presentation of intracellular pathogens or necrotic cells on MHC class I, thereby activating cytotoxic CD8^+^ T cells [17]. cDC1s have therefore attracted much interest in the context of cancer immunotherapy [18].

Owing to their multifaceted properties, BDCs are considered to induce a greater diversity of immune responses compared to moDCs when used in vaccination approaches. The first clinical trials evaluating pDCs and cDCs in solid tumors have already demonstrated safety and, to some extent, induction of tumor-specific immune responses. Tick-borne encephalitis vaccine-activated pDCs demonstrated a mature phenotype and produced large amounts of type I IFNs in melanoma patients, inducing tumor-specific immune responses [19]; this is in contrast to antigen-loaded, but nonactivated cDC2s administered to prostate cancer patients [20]. In a clinical trial of GM-CSF-activated cDC2s, three of 14 metastatic melanoma patients exhibited functional tumor-specific T cells. However, the authors postulated that immune responses might be optimized by a better-tailored activation stimulus [21]. In line with this hypothesis, we evaluated different protocols for in vitro activation of BDCs based on TLR ligands.

Here, we report the first protocol for achieving simultaneous in vitro activation of all BDC subsets based on activation of TLR3 and TLR7/8 with polyinosinic:polycytidylic acid [poly(I:C)] and R848 (Resiquimod), respectively. Our data suggest this to be the optimal combination for inducing a positive co-stimulatory profile in all BDC subsets, high secretion of IFN-α, and maximal secretion of IL-12p70. Activating all BDC subpopulations together increased immunostimulatory capacities compared to separately activating pDCs and cDCs with tailored protocols, indicating a synergistic cross-talk between BDC subsets during activation. Moreover, we demonstrate that activation of BDCs with this protocol results in enhanced migration, high NK-cell activation, and potent antigen-specific T-cell induction.

## Methods

### Healthy donors

Heparinized peripheral blood was collected from healthy donors after informed consent in accordance with the Declaration of Helsinki and approval by the Institutional Review Board of the Ludwig-Maximilians-Universität (Munich, Germany).

## Isolation and culturing of BDCs

BDCs were isolated from peripheral blood mononuclear cells (PBMCs) using the human Blood Dendritic Cell Isolation Kit II (Miltenyi Biotec, Bergisch Gladbach, Germany) and cultured in DC medium [X-Vivo 15 medium (Lonza, Verviers, Belgium), 2% human serum (HS; Sigma–Aldrich, Steinheim, Germany), 800 U/mL GM-CSF, 10 ng/mL IL-3 (both Peprotech, Rocky Hill, NJ, USA)].

Unless otherwise indicated, BDCs were activated for 20 h with 0.5 µM CpG (ODN 21,798, Miltenyi Biotec), 5 µg/mL R848, 25 µg/mL poly(I:C)-LMV (both Invivogen, Toulouse, France), 5 ng/mL IFN-γ (Peprotech), 2 µg/mL CD40L (BioCat, Heidelberg, Germany), 5 µg/mL protamine-RNA or combinations thereof.

Protamine-RNA complexes were formed as previously described [22] using mMESSAGE mMACHINE T7 Transcription Kit (Thermo Fisher Scientific, Vilnius, Lithuania) for mRNA generation from the supplied control template.

## Flow cytometry and cytokine quantification

Expression of immune checkpoint molecules on BDCs was assessed using Aqua-LIVE/DEAD (Life Technologies, Eugene, OR, USA) and the antibodies and isotype controls listed in Table 1. Median fluorescence intensity (MFI) ratios were calculated based on respective isotype controls.

**Table 1:** Antibodies used for characterization of BDC immune checkpoints

Type	Antigen	Dye	Clone	Manufacturer
Lineage markers	CD3	VioGreen	REA613	Miltenyi Biotec
	CD11c	PE/Vio770	REA618	Miltenyi Biotec
	CD14	VioGreen	REA599	Miltenyi Biotec
	CD19	VioGreen	REA675	Miltenyi Biotec
	CD123	APC/Vio770	REA918	Miltenyi Biotec
	CD141	PerCP/Vio700	REA674	Miltenyi Biotec
Immune checkpoints	CD40	APC	REA733	Miltenyi Biotec
	CD70	FITC	REA292	Miltenyi Biotec
	CD80	PE	REA661	Miltenyi Biotec
	CD86	VioBlue	REA968	Miltenyi Biotec
	CD197	APC	REA546	Miltenyi Biotec
	CD252	PE	11C3.1	Biolegend
	CD270	PE	RE247	Miltenyi Biotec
	CD273	FITC	RE985	Miltenyi Biotec
	CD274	BV421	MIH3	Biolegend
	CD275	PE	REA991	Miltenyi Biotec
	CD276	FITC	REA1094	Miltenyi Biotec
	CD279	BV421	EHA12.2H7	Biolegend
	B7-H4	FITC	MIH43	AbD Serotec
	B7-H5	APC	730,804	R&D
	Gal-9	FITC	REA435	Miltenyi Biotec
	GITRL	APC	REA841	Miltenyi Biotec
	HLA-DR	VioBlue	REA968	Miltenyi Biotec
Isotype controls	mIgG1	BV421	MOPC-21	Biolegend
	mIgG1	FITC	MOPC-21	Biolegend
	mIgG1	PE	MOPC-21	Biolegend
	mIgG2b	APC	MPC-11	Biolegend
	REA control (S)	APC	REA293	Miltenyi Biotec
	REA control (S)	FITC	REA293	Miltenyi Biotec
	REA control (S)	PE	REA293	Miltenyi Biotec
	REA control (S)	VioBlue	REA293	Miltenyi Biotec

Cytokine concentrations were quantified using the human MACSPlex Cytokine 12 Kit (Miltenyi Biotec).

Flow cytometry measurements were performed on a CytoFLEX S (Beckman Coulter, Krefeld, Germany). Data were analyzed using FlowJo v10.7 (BD, Ashland, OR, USA) and Prism v9.0.1 (GraphPad Software, San Diego, CA, USA). All stated values are reported as mean ± SEM.

## Isolation and culturing of pDCs and cDCs

pDCs were isolated from PBMCs by using the human CD304 MicroBead Kit, followed by isolation of cDCs from the flow-through by using the human Myeloid Dendritic Cell Isolation Kit (both Miltenyi Biotec). Alternatively, cDCs were isolated from whole PBMCs. pDCs were cultured for 16 h at 37 °C in DC medium, followed by stimulation with CpG for 3 h. cDCs were resuspended in DC medium and stimulated for 20 h with R848 and poly(I:C) immediately after isolation.

## T-cell stimulation assay

Activated BDCs, cDCs, pDCs, or a 1:1 mixture of cDCs and pDCs were pulsed with a cytomegalovirus (CMV), Epstein–Barr virus (EBV), influenza, and tetanus (CEFT) peptide pool (0.25 µg/mL/peptide, JPT, Berlin, Germany) for 2 h. T cells were isolated from PBMCs using human CD3 MicroBeads (Miltenyi Biotec) and stained using CellTrace Far Red (Life Technologies). DCs and autologous T cells were co-cultured at a ratio of 1:10 in R10 medium [RPMI-1640 (PAN-Biotech, Aidenbach, Germany), 10% fetal bovine serum, penicillin–streptomycin–glutamine (both Life Technologies)] for 5 days and subsequently stained for CD3 (PerCP/Vio700, REA613), CD4 (APC/Vio770, REA623), and CD8 (PE/Vio770, REA734) (all Miltenyi Biotec).

## Migration assay

The lower part of a 96-transwell plate (5 µm pore size, Corning, Kennebunk, ME, USA) was filled with 200 µL X-Vivo15 medium supplemented with 2% HS and either 200 ng/mL CCL19 (R&D Systems), 200 ng/mL CCL21 (Biolegend) or no chemokine. BDCs were seeded in the upper chamber in technical duplicates. Cells were harvested after 3 h incubation at 37 °C from both chambers, stained for CD11c and CD123, and analyzed by flow cytometry together with Precision Count Beads (Biolegend).

## NK-cell activation assay

NK cells were isolated from fresh PBMCs using the human NK Cell Isolation Kit (Miltenyi Biotec). BDCs were co-cultured with autologous NK cells for 24 h at a ratio of 1:10 in R10 medium. NK-cell activation was assessed by staining for CD69 (APC, FN50) or mIgG1 isotype (APC, MOPC-21) on cells positive for CD16 (FITC, 3G8) and CD56 (PE, MEM-188; all Biolegend).

## Expansion of antigen-specific T cells

CD8^+^ T cells were isolated using CD8 T Cell Isolation Kit (Miltenyi Biotec) and cultured in T-cell medium (X-Vivo 15 medium, 5% HS, penicillin–streptomycin–glutamine) supplemented with 5 ng/mL IL-7 (Peprotech) for 20 h. Autologous BDCs, activated with R848 + poly(I:C), were pulsed for 2 h with 1 µg/mL/peptide of PepTivator CEF MHC Class I Plus, WT1, or SARS-CoV-2 Prot_M, Prot_N and Prot_S (all Miltenyi Biotec). T cells and pulsed BDCs were co-cultured at a T cell/BDC ratio of 4:1 in T-cell medium containing 30 ng/mL IL-21 (Peprotech). On days 3, 5, and 7, co-cultures were expanded 1:1 with medium containing 10 ng/mL IL-7 and IL-15 (Peprotech). Antigen-specific T-cell expansion was monitored after 10 days by restimulation for 4 h with equal numbers of autologous PBMCs and 1 µg/mL peptides in medium containing 25 µM monensin and 10 µg/mL brefeldin A (both Sigma–Aldrich). Cells were stained for CD3 (FITC, UCHT1, Biolegend) and CD8 (VioBlue, REA734, Miltenyi Biotec). The percentage of cytokine-secreting CD8^+^ T cells was analyzed by intracellular staining for IFN-γ (PE, B27) and TNF-α (APC, MAb11; both Biolegend).

## Results

### Isolated BDCs express positive immune checkpoint molecules

**Fig. 1:**
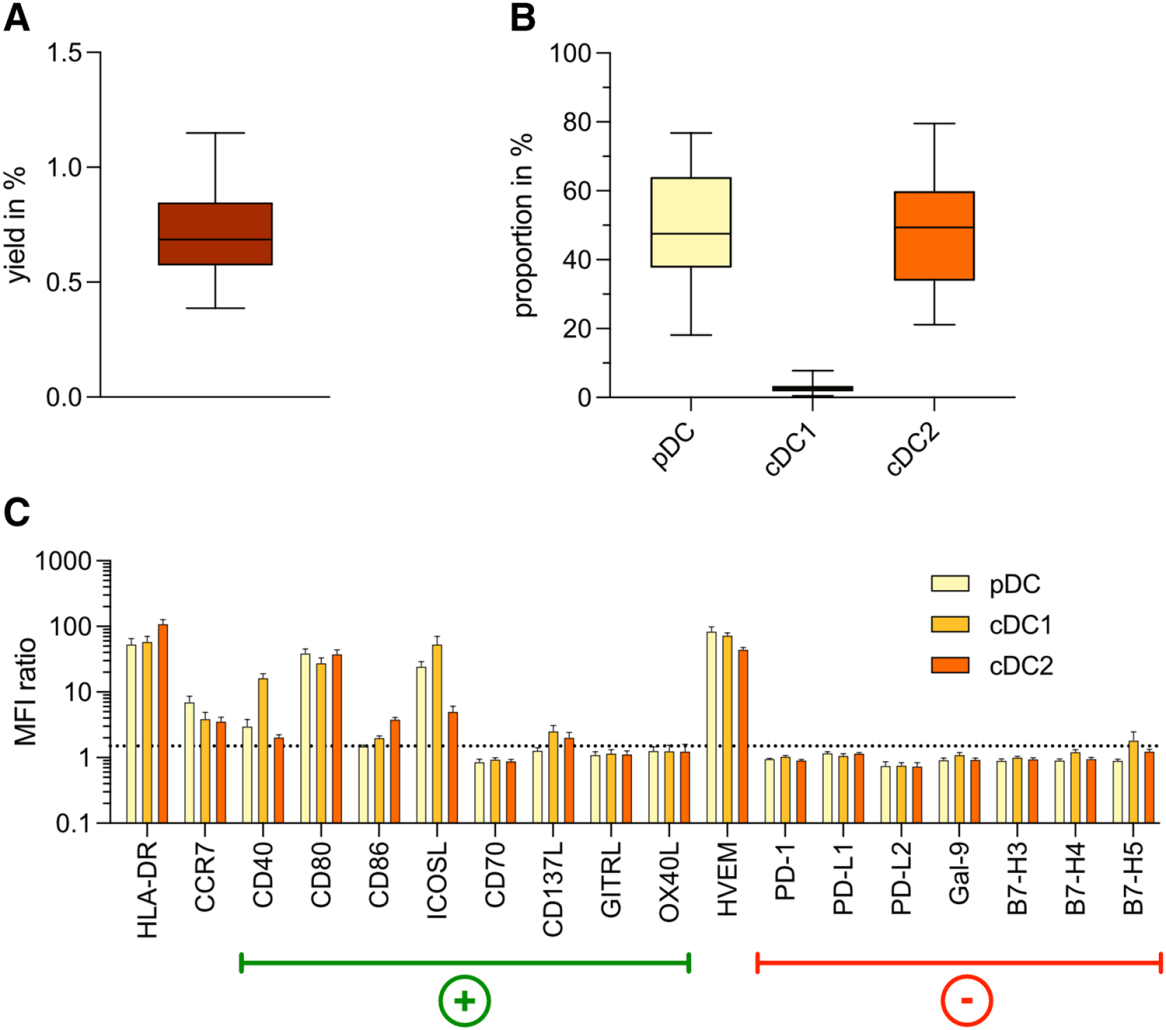
Characterization of BDCs isolated from peripheral blood. **a** Yield of BDCs isolated from healthy donor PBMCs (*n* = 30). **b** Proportion of DC subsets among isolated BDCs (*n* = 18). **c** MFI ratios of co-stimulatory and co-inhibitory immune checkpoints expressed by pDCs (light yellow), cDC1s (light orange), and cDC2s (dark orange) isolated from peripheral blood (*n* = 6). Bars represent mean ± SEM. Box-and-whisker plots show 5th and 95th percentile

BDCs were isolated from PBMCs with a median yield of 0.69% (Fig. 1a). The expression of surface markers by BDCs was analyzed by flow cytometry based on the gating strategy shown in Supplementary Fig. 1. The median relative frequencies of pDCs, cDC1s, and cDC2s among BDCs were 47.6%, 2.6%, and 49.4%, respectively (Fig. 1b).

Analysis by flow cytometry and calculation of MFI ratios revealed that all BDC subsets expressed HLA-DR and several positive co-stimulatory immune checkpoint molecules. All subsets expressed high levels of CD80 (pDC: 38.8 ± 6.3; cDC1: 27.5 ± 5.4; cDC2: 37.7 ± 6.4), whereas CD86 was only weakly expressed on cDC1s and cDC2s, and absent on pDCs. ICOSL was highly expressed by pDCs (24.4 ± 4.8) and cDC1s (53.1 ± 17.2), whereas CD40 was present predominantly on cDC1s (16.3 ± 2.7). Furthermore, BDCs expressed no or only minimal levels of the immune checkpoint molecules CD70, CD137L, GITRL, and OX40L. We detected no expression of co-inhibitory markers, including PD-L1, PD-L2, Gal-9, B7-H3, B7-H4, and B7-H5. All BDC subsets expressed high levels of HVEM (pDC: 83.8 ± 14.3; cDC1: 73.0 ± 6.8; cDC2: 44.1 ± 3.3). The migratory receptor CCR7 was weakly expressed on all subsets, with its highest expression on pDCs (7.0 ± 1.6) (Fig. 1c).

## A combination of TLR ligands is required to induce a positive co-stimulatory profile on all BDCs

**Fig. 2:**
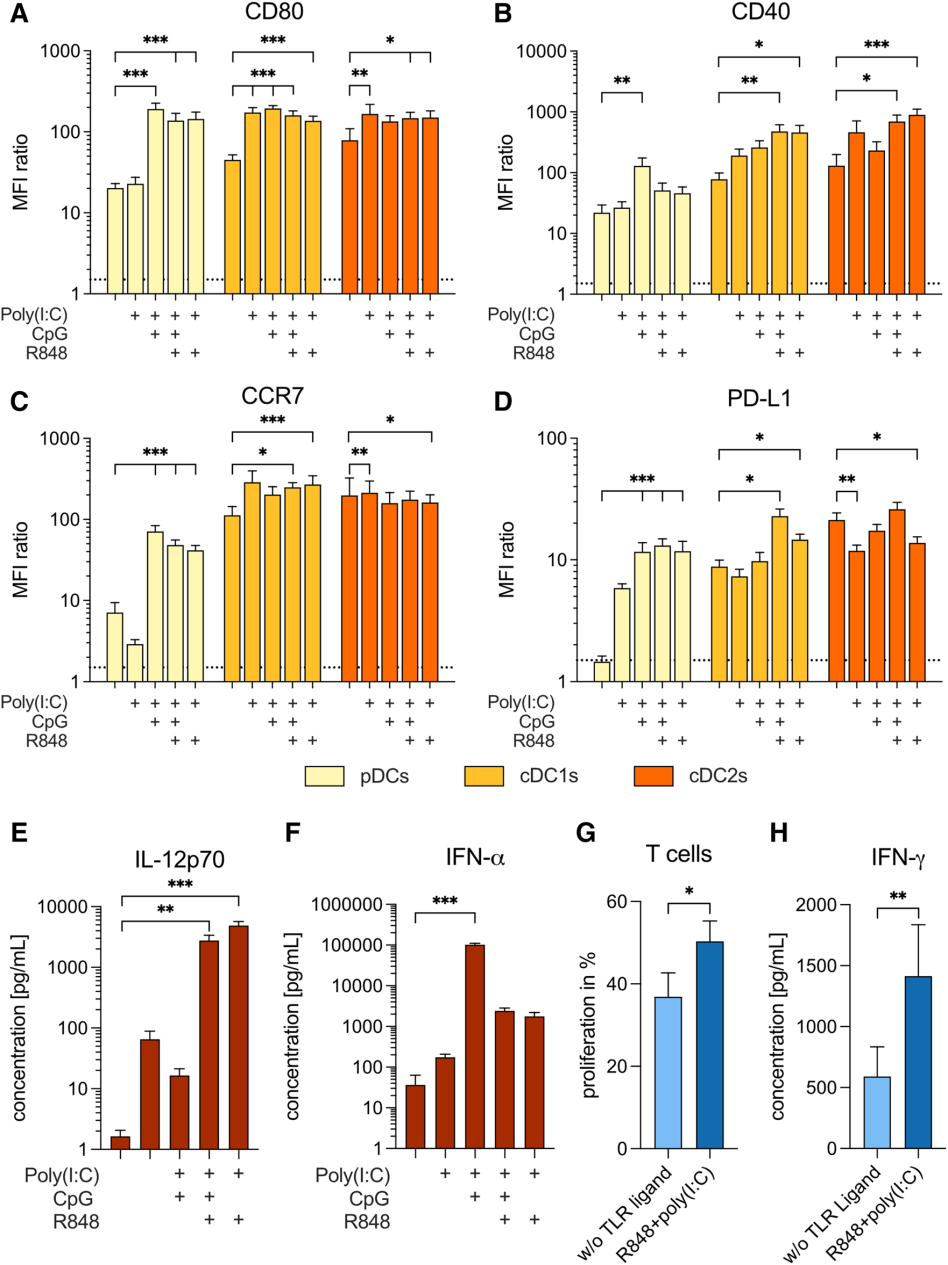
Comparison of TLR ligand cocktails for simultaneous activation of all BDC subsets. Expression of CD80 (**a**), CD40 (**b**), CCR7 (**c**), and PD-L1 (**d**) on BDC subsets, and secretion of IL-12p70 (**e**) and IFN-α (**f**) by BDCs in response to activation with different TLR ligand cocktails (*n* = 6). (**g**) T-cell proliferation and (**h**) secretion of IFN-γ by T cells induced by BDCs activated without TLR ligands (light blue) or R848 + poly(I:C) (dark blue) (*n* = 14–16). Bars represent mean ± SEM. Statistical differences compared to the control without TLR ligand were analyzed by paired one-way ANOVA with Bonferroni's multiple comparison test (**a–f**) or Wilcoxon matched-pairs signed-rank test (**g, h**). Only significant differences are shown: *p < 0.05, **p < 0.01, ***p < 0.001

To identify a protocol for activating all BDC subsets simultaneously, we analyzed the expression of immune checkpoint molecules by BDCs in response to different TLR ligands and combinations thereof (Fig. 2a–d). Supplementary Table 1 reports expression of the immune checkpoint molecules CD80, CD40, PD-L1, and the migratory receptor CCR7 for all BDC subsets in response to different combinations of TLR ligands. Activation with TLR3 ligand poly(I:C) caused high expression of CD80 and CD40 and the highest expression of CCR7 on cDC1s and cDC2s. However, expression of CD80 and CD40 was further increased on cDC subsets if poly(I:C) was combined with other TLR ligands. The combination of poly(I:C) with the TLR9 ligand CpG consistently caused the highest expression of these markers on pDCs (CD80: 191.1 ± 34.8; CD40: 129.2 ± 44.7; CCR7: 70.9 ± 13.2). The combination of poly(I:C) with the TLR7/8 ligand R848 resulted in consistently high expression of CD80, CD40, and CCR7 on all BDC subsets (Fig. 2a–c).

The co-inhibitory immune checkpoint PD-L1 was not expressed on pDCs and only weakly expressed by cDC1s and cDC2s cultured without TLR ligands. Activation with CpG + R848 + poly(I:C) led to the highest expression of PD-L1 on all BDC subsets (pDCs: 13.1 ± 1.8; cDC1s: 22.9 ± 3.3; cDC2s: 26.0 ± 3.6), whereas R848 + poly(I:C) caused only intermediate levels of expression (pDCs: 11.8 ± 2.4; cDC1s: 14.7 ± 1.6; cDC2s: 13.8 ± 1.7) (Fig. 2d).

## TLR stimulation of BDCs results in distinct cytokine secretion

Next, we evaluated the secretion of cytokines by BDCs in response to TLR activation. We observed the highest secretion of IL-12p70 by simultaneous triggering of TLR3 and TLR7/8 with R848 + poly(I:C). Interestingly, adding CpG led to significantly reduced IL-12 levels (4.9 × 10^3^ ± 8.2 × 10^2^ vs. 2.8 × 10^3^ ± 6.2 × 10^2^ pg/mL; *p* = 0.014). Using poly(I:C) ± CpG caused intermediate IL-12 levels (Fig. 2e). Stimulation with only R848 or CpG or the combination of the two did not lead to IL-12 production in a parallel study, nor did protamine-RNA (Supplementary Fig. 2a). Addition of IFN-γ, but not CD40L, to the combination of R848 and poly(I:C) further enhanced secretion of IL-12 (Supplementary Fig. 3a).

By contrast, we observed the highest IFN-α concentrations using poly(I:C) + CpG (Fig. 2f). Adding R848 to this combination caused a significant reduction of IFN-α levels (1.0 × 10^5^ ± 8.3 × 10^3^ pg/mL vs. 2.4 × 10^3^ ± 4.2 × 10^2^; *p* < 0.001), similar to activating with R848 + poly(I:C) (1.8 × 10^3^ ± 4.4 × 10^2^ pg/mL). Poly(I:C) induced only negligible IFN-α secretion. Activation with CpG, R848, or protamine-RNA caused notable IFN-α secretion (Supplementary Fig. 2b). Addition of IFN-γ or CD40L to the combination of R848 and poly(I:C) did not change the IFN-α response (Supplementary Fig. 3b).

Since R848 + poly(I:C) caused an overall positive co-stimulatory expression on BDCs, high IL-12 secretion, and intermediate levels of IFN-α secretion, we focused on this combination for subsequent experiments.

## Activation of BDCs with R848 + poly(I:C) increases T-cell responses

Next, we measured the T-cell responses induced by TLR-activated BDCs. To do so, we co-cultured BDCs with autologous T cells and measured T-cell proliferation and IFN-γ secretion after five and four days, respectively. Nonactivated BDCs induced a T-cell proliferation of 36.9 ± 5.8%. This was significantly increased to 50.3 ± 5.0% (*p* = 0.018) if BDCs were activated with R848 + poly(I:C) (Fig. 2g). Similarly, T cells secreted significantly higher amounts of IFN-γ in response to R848 + poly(I:C)-activated BDCs compared to nonactivated BDCs (1.4 × 10^3^ ± 4.2 × 10^2^ vs. 5.9 × 10^2^ ± 2.4 × 10^2^ pg/mL; *p* = 0.008) (Fig. 2h).

## IL-12 and IFN-α are secreted at different time points

To determine when cytokines are secreted by BDCs, we analyzed cytokine secretion 1, 3, 6, and 20 h after TLR activation. Owing to time constraints, we introduced an additional resting step of 16 h between BDC isolation and activation. As a control, we also evaluated a 20-h activation without the resting step.

**Fig. 3:**
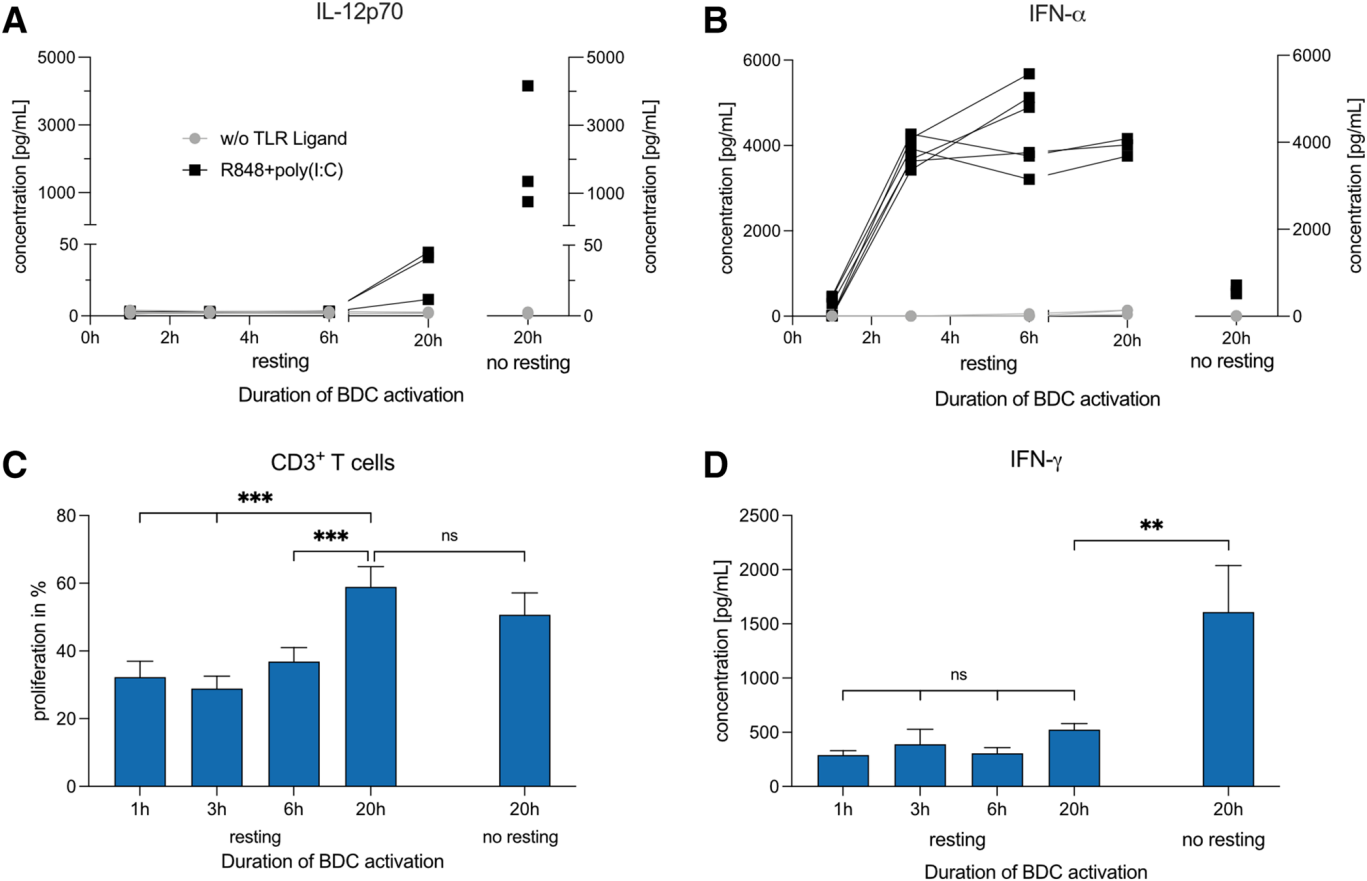
Evaluation of shorter durations of BDC activation for differences in cytokine secretion and induction of T-cell responses. Cumulative secretion of IL-12p70 (**a**) and IFN-α (**b**) by BDCs after 1, 3, 6, and 20 h of activation with R848 and poly(I:C). BDCs were "rested" for 16 h before the TLR ligand was added. In addition, BDCs were activated directly after isolation for 20 h (*n* = 3–6). **c** Induction of T-cell proliferation by BDCs activated for various time periods (*n* = 9). **d** Secretion of IFN-γ in co-cultures of T cells and BDCs that were activated for various periods of time (*n* = 6). Bars represent mean ± SEM. Statistical differences compared to 20-h activation with TLR ligands after resting for 16 h were analyzed by paired one-way ANOVA with Bonferroni's multiple comparison test. Only significant differences are shown: **p < 0.01, ***p < 0.001

We observed no secretion of IL-12 within the first 6 h of activation and only a small response after 20 h (32.2 ± 10.4 pg/mL). In contrast, when we added the R848 + poly(I:C) directly after cell isolation, we observed strong IL-12 secretion for the same donors (2.1 × 10^3^ ± 1.1 × 10^3^ pg/mL) (Fig. 3a). For IFN-α, as early as 3 h after adding R848 + poly(I:C), we measured a mean concentration of 3.8 × 10^3^ ± 1.3 × 10^2^ pg/mL that remained comparable after 6 and 20 h. Activation for 20 h directly after isolation resulted in lower IFN-α secretion compared to no resting after 20 h (4.0 × 10^3^ ± 1.2 × 10^2^ vs. 6.0 × 10^2^ ± 5.9 × 10^1^ pg/mL) (Fig. 3b).

## A shorter BDC activation time does not increase T-cell responses

To determine if a shorter duration of BDC activation improves T-cell responses, we prepared co-cultures of T cells and autologous BDCs activated with R848 + poly(I:C) for 1, 3, 6, and 20 h after 16 h "resting" or for 20 h directly after cell isolation. We analyzed T-cell proliferation and IFN-γ secretion after five and four days, respectively. BDCs activated for 20 h induced a T-cell proliferation of 59.0 ± 6.0%. This was significantly higher compared to shorter activation periods of between 1 and 6 h (range: 32.3–36.9%; *p* < 0.001). "Resting" versus "no resting" prior to 20-h activation did not result in a significant difference (*p* = 0.781) (Fig. 3c). In contrast, maximum IFN-γ secretion by T cells was detected with BDCs activated for 20 h directly after cell isolation (1.6 × 10^3^ ± 4.3 × 10^2^ pg/mL), compared to BDCs rested for 16 h (for 20 h: 5.2 × 10^2^ ± 5.6 × 10^1^ pg/mL; *p* = 0.007). No significant differences in IFN-γ secretion between BDCs activated for 1, 3, 6, or 20 h after the resting step was observed (Fig. 3d).

## Individual activation of cDCs and pDCs with a tailored protocol is not superior to a combined activation of all BDCs

**Fig. 4:**
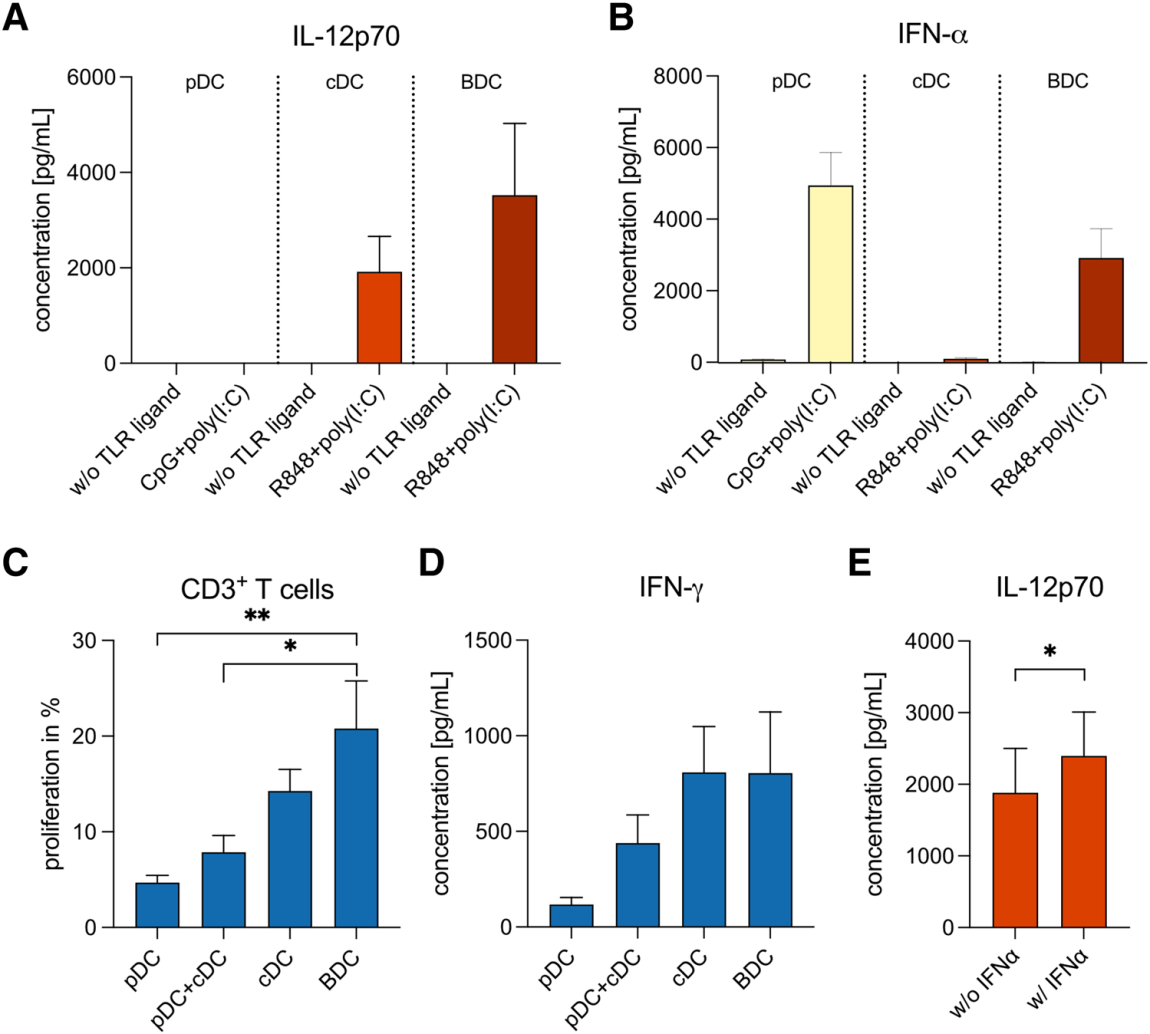
Comparison between tailored activation protocols for cDCs and pDCs and simultaneous activation of BDCs. **a, b** Secretion of IL-12p70 and IFN-α by pDCs, cDCs, and BDCs upon activation with tailored protocols for TLR stimulation (*n* = 6). **c, d** T-cell proliferation and IFN-γ secretion induced in co-culture experiments with autologous pDCs, cDCs, BDCs, or a 1:1 mixture of pDCs and cDCs (*n* = 6). Bars represent mean ± SEM. Statistical test: paired one-way ANOVA with Bonferroni's multiple comparison test; *p < 0.05, **p < 0.01. **e** Effect of IFN-α addition on IL-12p70 secretion by cDCs activated with R848 + poly(I:C) (*n* = 10). Bars represent mean ± SEM. Statistical test: Wilcoxon matched-pairs signed-rank test, *p < 0.05

An important translational question is whether separate activation of cDCs and pDCs with a tailored activation protocol is superior to the combined activation of all BDC subsets. To address this question, we isolated pDCs, cDCs, and entire BDCs from the same donors and activated cDCs and BDCs with R848 + poly(I:C) for 20 h immediately after isolation. By contrast, pDCs were rested for 16 h before exposing them to CpG + poly(I:C) for 3 h, as described for optimal IFN-α secretion above. Secretion of IL-12 was higher by BDCs than by cDCs (3.5 × 10^3^ ± 1.5 × 10^3^ and 1.9 × 10^3^ ± 7.4 × 10^2^ pg/mL, respectively). Activation with CpG + poly(I:C) resulted in strong secretion of IFN-α by pDCs (4.9 × 10^3^ ± 9.2 × 10^2^ pg/mL), while observed cytokine concentrations for R848 + poly(I:C)-activated BDCs were lower (2.9 × 10^3^ ± 8.1 × 10^2^ pg/mL). We did not observe any cytokine secretion in controls without TLR ligands (Fig. 4a, b).

In co-culture experiments of autologous T cells and BDCs, pDCs, cDCs, or a 1:1 mixture of pDCs and cDCs, T-cell proliferation was significantly higher when using BDCs collectively activated, compared to a mixture of separately activated pDCs and cDCs (20.8 ± 5.0% vs. 7.9 ± 1.7%, *p* = 0.044) (Fig. 4c). Similarly, IFN-γ secretion by T cells was highest in response to BDCs (8.0 × 10^2^ ± 3.2 × 10^2^ pg/mL), whereas the mixture of pDCs and cDCs exhibited a 1.8-fold reduction (4.4 × 10^2^ ± 1.5 × 10^2^ pg/mL) (Fig. 4d).

IFN-α supplementation of cDCs activated with R848 + poly(I:C) significantly increased IL-12 secretion (2.4 × 10^3^ ± 6.1 × 10^2^ vs. 1.9 × 10^3^ ± 6.2 × 10^3^ pg/mL; *p* = 0.040) (Fig. 4e).

## TLR-activated BDCs expand antigen-specific CD8^+^ T cells

**Fig. 5:**
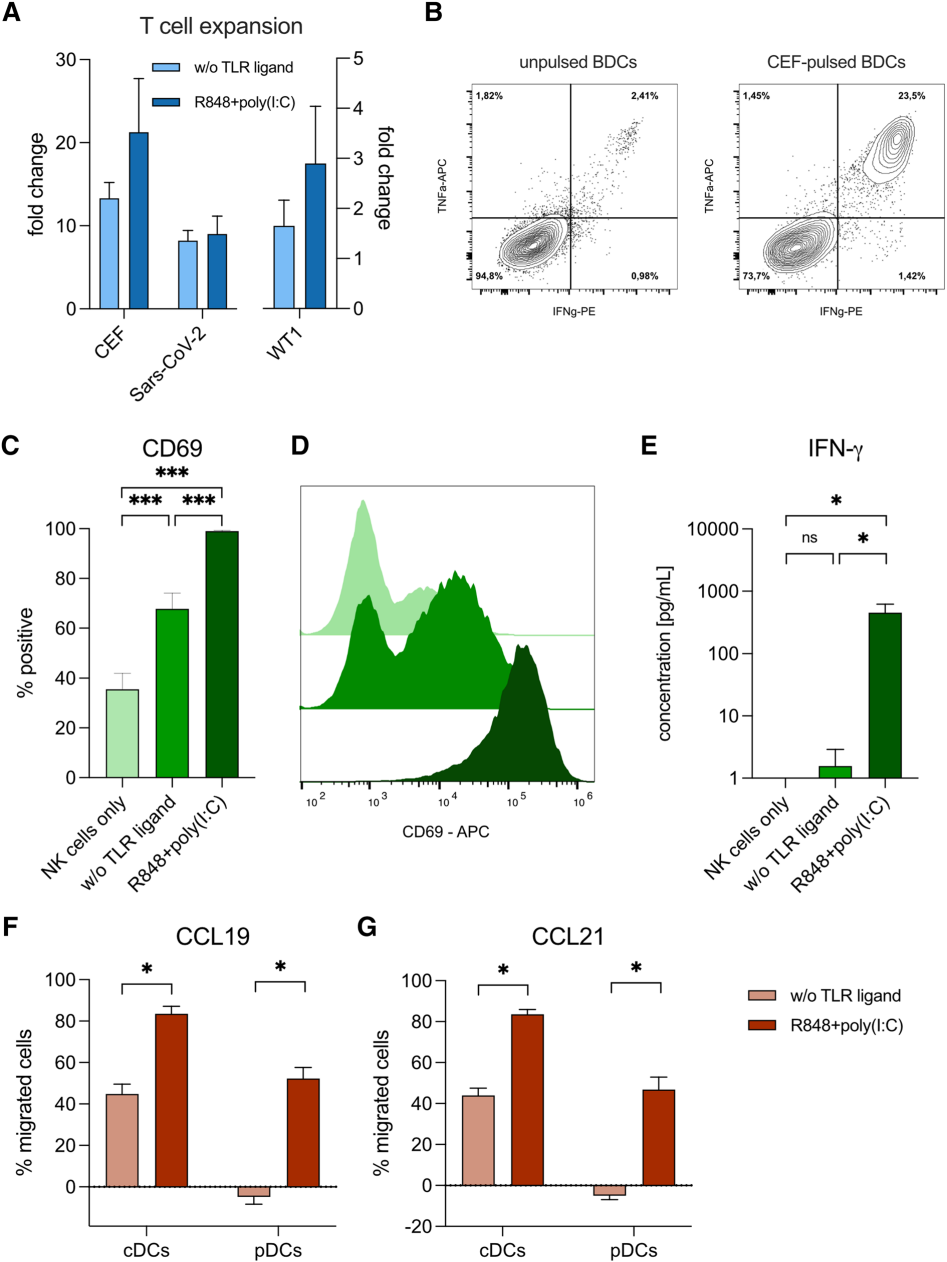
Migration, NK-cell activation, and T-cell expansion by BDCs. **a** Expansion of T cells specific for CEF, WT1, and SARS-CoV-2 by BDCs activated with TLR ligands (*n* = 3–4). **b** Representative flow cytometry analysis of antigen-specific T-cell expansion by BDCs activated with R848 + poly(I:C). BDCs were either not pulsed (left) or pulsed with peptides (right) prior to co-culturing with autologous T cells. **c** Activation of NK cells in co-cultures with autologous BDCs activated with TLR ligands (*n* = 9). **d** Representative example for NK-cell activation by BDCs. Color coding: NK cells only (light green), BDCs activated without TLR ligands (green) and with R848 + poly(I:C) (dark green). **e** Secretion of IFN-γ in co-cultures of NK cells and autologous BDCs (*n* = 7). Migration of BDC subsets towards CCL-19 (**f**) and CCL-21 (**g**) upon activation with TLR ligands in a transwell assay (*n* = 6). Bars represent mean ± SEM. Statistical tests: paired one-way ANOVA with Bonferroni's multiple comparison test (**c**, **e**), Wilcoxon matched-pairs signed-rank test (**f**, **g**), *p < 0.05, ***p < 0.001

In order to test the capacity of BDCs to induce antigen-specific T-cell responses, autologous CD8^+^ T cells were co-cultured with BDCs pulsed with viral peptides derived from CMV, EBV, and influenza A (CEF peptides) or SARS-CoV-2, or peptides derived from the tumor oncogene WT1 (Fig. 5a, b).

R848 + poly(I:C)-activated BDCs demonstrated enhanced expansion of CEF-specific CD8^+^ T cells, compared to nonactivated BDCs (mean fold change of antigen-specific T cells compared to respective controls with unpulsed BDCs: 21.3 ± 6.5 vs. 13.3 ± 1.9; ± SEM). Similarly, expansion of T cells specific for SARS-CoV-2 from donors that had been previously infected by the virus was higher by R848 + poly(I:C)-activated BDCs compared to nonactivated BDCs (9.0 ± 2.1 vs. 8.2 ± 1.2). For WT1-specific T cells, R848 + poly(I:C)-activated BDCs similarly caused an improved expansion of antigen-specific T cells compared to non-activated BDCs (2.9 ± 1.1 vs. 1.6 ± 0.5).

## BDCs activated with R848 + poly(I:C) results in increased NK-cell activation

We tested the activation of NK cells co-cultured with autologous BDCs. Co-cultivation with nonactivated BDCs resulted in an increased frequency of CD69-expressing NK cells compared to controls with only NK cells (70.7 ± 6.1 vs. 41.6 ± 7.9%). Addition of R848 + poly(I:C)-activated BDCs led to CD69 expression by almost all NK cells (99.1 ± 0.2%; *p* < 0.001) at very high levels (Fig. 5c, d). Similarly, TLR-activated BDCs resulted in a significant increase of IFN-γ secretion by NK cells compared to nonactivated BDCs (4.5 × 10^2^ ± 1.7 × 10^2^ vs. 1.6 ± 1.3 pg/mL; *p* = 0.019) (Fig. 5e).

## R848 + poly(I:C) increases specific migration of BDCs

Finally, we analyzed whether R848 + poly(I:C) increased the specific migration of DCs toward the chemokines CCL-19 and CCL-21. pDCs migrated toward CCL-19 only upon activation with R848 + poly(I:C) (52.3 ± 5.4%). For cDCs, TLR-activation increased migration from 44.9 ± 4.8% to 83.5 ± 3.6% (*p* = 0.031) upon TLR activation. Similar results were obtained for CCL-21 (Fig. 5f, g).

## Discussion

BDCs have become a promising alternative to moDCs in cancer immunotherapies owing to their multifaceted properties. However, results from first clinical trials indicate the need for improved activation protocols. Here, we systematically established a protocol for the simultaneous activation of all BDC subsets *in vitro* using a combination of two TLR ligands. Although BDCs represent only about 1% of all PBMCs, automated systems allow their rapid isolation from buffy coats or leukapheresis products to high purity in a closed system [23].

We observed low to intermediate expression of positive co-stimulatory immune checkpoint molecules on BDCs after isolation from peripheral blood. Stimulation with TLR ligands induced high expression of the co-stimulatory molecules CD80 and CD40 and the migratory receptor CCR7, whereby a combination of a TLR3 with a TLR7/8 ligand proofed to be optimal for all BDC subsets. We also observed upregulation of PD-L1 in all BDC subsets, providing the rationale to combine BDC vaccination with PD-1/PD-L1 blockade. This has already been successfully evaluated in pre-clinical studies, however, results from clinical trials are scarce [24–26]. Nevertheless, the feasibility of a similar approach has been demonstrated in metastatic melanoma patients by blockade of CTLA-4 in combination with moDCs [27].

TLR ligands also caused strong secretion of IL-12 and IFN-α by BDCs. Whereas IFN-α secretion was at its highest level upon activation with CpG + poly(I:C), IL-12 secretion was maximal with R848 + poly(I:C). In contrast, single TLR stimulation-induced generally lower BDC responses. This is in line with previous results, describing TLR synergy between TLR3 or TLR4 and TLR8 in human moDCs and cDC2s resulting in potent IL-12p70 induction [28]. Addition of IFN-γ to the combination of R848 and poly(I:C) led to further enhanced secretion of IL-12p70, similar to previous results in moDCs [28].

We decided to focus on R848 + poly(I:C) to optimize Th1 differentiation, which is induced by IL-12. Similar to our results, R848 + poly(I:C) has been reported to upregulate co-stimulatory immune checkpoints in BDC subsets and to induce cytokine and chemokine secretion (i.e., type I IFNs, IL-12) in a humanized mouse model [29]. Addition of CpG to R848 + poly(I:C) provided no benefit with respect to immune checkpoint expression and cytokine secretion by BDCs. This reflects the previous finding that single-stranded DNA oligonucleotides such as CpG inhibit the activation of moDCs and nonhematopoietic cells by poly(I:C) [30].

By analyzing the kinetics of cytokine secretion, we found that IFN-α production by BDCs starts only a few hours after stimulation with R848 + poly(I:C), whereas the onset of IL-12 secretion appears to be later. This is consistent with results from a humanized mouse model [29]. Unexpectedly, the introduction of a resting step before TLR activation of BDCs strongly increased IFN-α secretion, whereas IL-12 was reduced. Spontaneous apoptosis of cDCs during ex vivo handling might explain this observation, whereas cultivation with IL-3 during the resting step might have promoted survival of pDCs. Conversely, preincubation with IL-3 for longer periods was reported to decrease the IFN-α secretion capacity of pDCs [31], however, as appropriate controls were missing in that study, spontaneous apoptosis might have been at play. Furthermore, we observed that reducing the time span of BDC activation and introducing the resting step resulted in reduced T-cell responses compared to BDC activation for 20 h immediately after their isolation.

Evaluation of tailored activation protocols for BDC subsets allowed us to elucidate the individual contributions of pDCs and cDCs to induce immune responses. cDCs induced stronger T-cell responses than pDCs, correlating with IL-12 secretion. The role assigned to pDCs in shaping the immune response in oncological malignancies remains controversial, with contradicting correlations between pDC infiltration and disease prognosis [32, 33]. Recently, the results of a clinical trial of protamine-RNA-activated pDCs, cDCs, or a combination of pDCs and cDCs in prostate cancer were reported. In all three treatment arms, antigen-specific T-cell responses were induced and correlated with radiographic progression-free survival, but no significant differences were observed [34]. Interestingly, when the authors used protamine-RNA for DC activation, cDCs did not secrete IL-12, but pDCs did, albeit at a low level. This might have been caused by a rare contaminating cDC population sharing common pDC markers [35], as the purity of the pDC vaccine was only modest. However, in our study, BDCs elicited higher immunostimulatory capacities than a combination of pDCs and cDCs activated with tailored protocols, underlining the rationale for activating all BDC subsets simultaneously.

DC vaccines aim to elicit and maintain T-cell responses against tumor-specific antigens. WT1 is a universal tumor target antigen due to its expression pattern in multiple different cancer entities, thus having great potential for developing immunotherapies [36]. However, inducing sufficient immune responses is a major challenge owing to the low immunogenicity of nonmutated tumor antigens and clonal deletion of self-reactive T cells [37]. R848 + poly(I:C)-activated BDCs were able to expand WT1-specific T cells, providing a rationale for their further development as an immunotherapeutic tool. In addition to their use as monotherapy, DC vaccines might enhance existing immune responses in the context of Chimeric antigen receptor (CAR) T-cell therapies. Accordingly, moDCs have been shown to boost WT1-specific CAR T cells in a humanized mouse model, resulting in enhanced inhibition of tumor growth [38].

DC vaccination trials to date have mainly focused on induction of T-cell responses, however, NK-cell activation by DCs constitutes an interesting upside for immunotherapies. Not only do NK cells recruit and activate further DCs, promoting T-cell responses, they can also eliminate tumor cells directly. Thus, tumor-cell material is released that can be processed by further DCs and be presented to T cells [39]. As activation of NK cells is mediated by IL-12 and type I interferons [40], BDCs activated with R848 + poly(I:C) demonstrated potent NK-cell activation with respect to CD69 expression and IFN-γ secretion. Importantly, NK-cell activation has been shown to correlate with clinical outcomes in several clinical trials, including those of a DC vaccine targeting WT1 in AML [39, 41, 42].

Migration of DCs to draining lymph nodes relies on the migratory receptor CCR7 and is central to induce adaptive immune responses. CCL-19 and CCL-21 promote the migration of DCs to the lymph nodes themselves and to the T-cell zone within the lymph nodes, enabling T-cell activation by DCs [43]. Only a few clinical trials have monitored the migration of their DC vaccine, revealing that only a small percentage of the injected DCs successfully migrated to the lymph nodes, underlining the need for enhanced DC migration capacities [44–46]. Activation with R848 + poly(I:C) upregulated expression of CCR7 on all BDCs. As a consequence, we observed improved migration of BDCs toward CCL-19 and CCL-21. Importantly, pDCs migrated only upon TLR activation.

Our investigations show R848 + poly(I:C) to be an optimized cocktail for ex vivo activation of all BDC subsets. Our findings support the further investigation and usage in early clinical trials – as standalone or in conjunction with other immunotherapeutic strategies including adoptive T-cell transfer and checkpoint inhibition.

### Supplementary Information

Below is the link to the electronic supplementary material.

Supplementary file1 (PDF 877 KB)

## Data Availability

All data generated and analyzed during the current study are available from the corresponding author on reasonable request.

## Abbreviations

AML: Acute myeloid leukemia
BDC: Blood dendritic cell
cDC: Conventional dendritic cell
CEF: Cytomegalovirus (CMV), Epstein–Barr virus (EBV), influenza
CEFT: Cytomegalovirus (CMV), Epstein–Barr virus (EBV), influenza, tetanus
CpG: CpG oligodeoxynucleotide
GM-CSF: Granulocyte–macrophage colony-stimulating factor
HS: Human serum
MFI: Median fluorescence intensity
moDC: Monocyte-derived dendritic cell
PBMC: Peripheral blood mononuclear cell
pDC: Plasmacytoid dendritic cell
poly(I:C): Polyinosinic:polycytidylic acid
R848: Resiquimod
TLR: Toll-like receptor
WT1: Wilms tumor 1
