# Supplementary material for: Blood DCs activated with R848 and poly(I:C) induce antigen-specific immune responses against viral and tumor-associated antigens
Source: Cancer Immunol Immunother. 2021 Nov 25;71(7):1705–18. doi: 10.1007/s00262-021-03109-w (PMC8614222; doi:10.1007/s00262-021-03109-w)
Supplement: Supplementary file 1 — Supplementary file1 (PDF 877 KB) [file 262_2021_3109_MOESM1_ESM.pdf]

# Supplementary Information

## Supplementary Figures

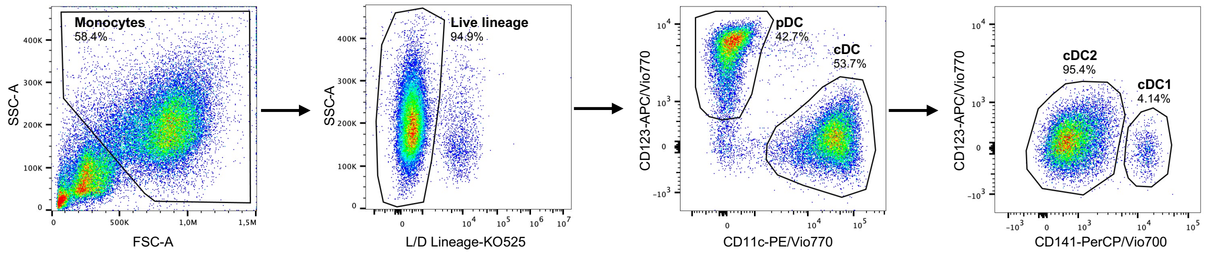


**Supplementary Figure 1: Gating strategy for BDCs.** Cells were gated for monocytes followed by exclusion of dead cells and cells positive for the lineage markers CD3, CD14, or CD19. pDCs and cDCs were subsequently characterized according to their expression of CD123 or CD11c, respectively. Based on their expression of CD141, cDCs were further subdivided into cDC1 and cDC2.

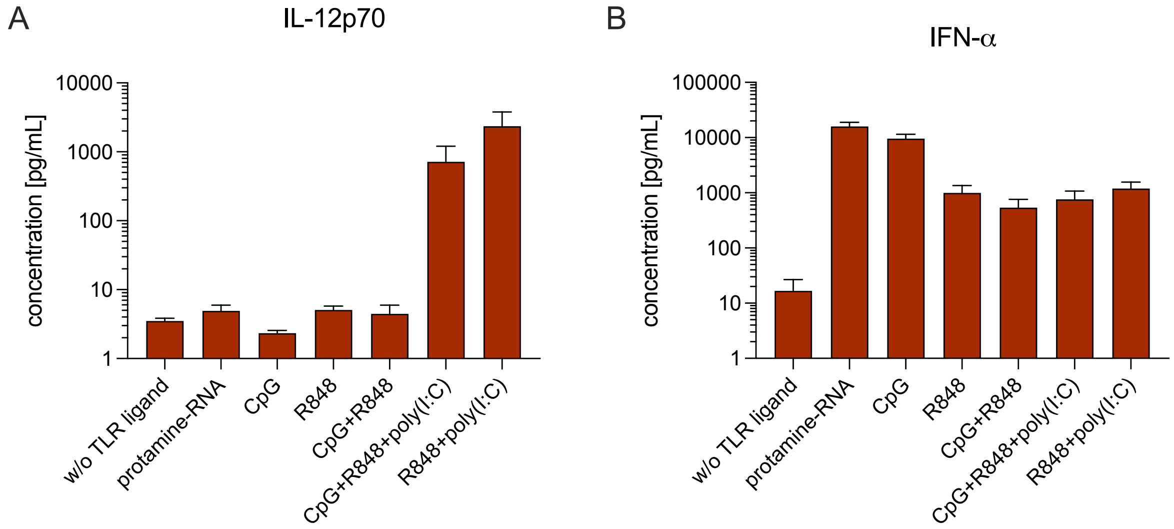


**Supplementary Figure 2: Cytokine secretion of BDCs induced by TLR activation.** Secretion of IL-12p70 (a) and IFN-α (b) induced by activation with different combinations of TLR ligands. Bars represent mean±SEM (*n*=6).

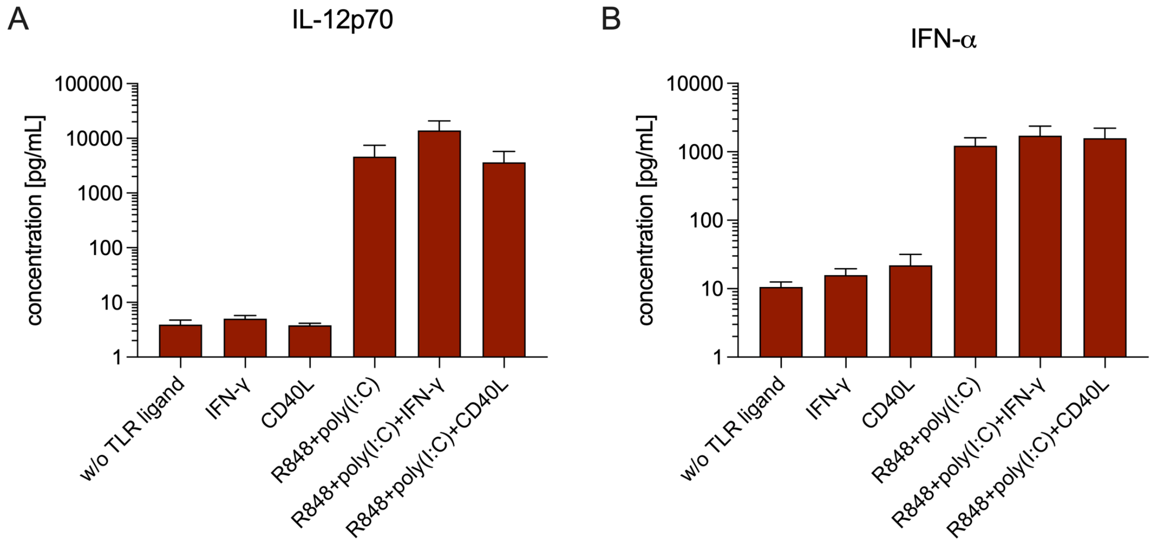


**Supplementary Figure 3: Amplification of BDC cytokine secretion induced by R848+poly(I:C) in combination with IFN-γ and CD40L.** Secretion of IL-12p70 (a) and IFN-α (b) induced by activation with IFN-γ, CD40L, R848+poly(I:C) or combinations thereof. Bars represent mean±SEM (*n*=6).

## Supplementary Table

**Supplementary Table 1:** Mean MFI ratios (±SEM) of CD80, CD40, CCR7, and PD-L1 expressed on pDC, cDC1, and cDC2 after 20h activation with different combinations of TLR ligands.

| | | CD80 | | CD40 | | CCR7 | | PD-L1 | |
|---|---|---|---|---|---|---|---|---|---|
| | | **mean** | **SEM** | **mean** | **SEM** | **mean** | **SEM** | **mean** | **SEM** |
| **pDC** | w/o TLR ligand | 20.21 | 2.78 | 22.04 | 7.33 | 7.11 | 2.30 | 1.46 | 0.16 |
| | poly(I:C) | 22.90 | 4.64 | 26.59 | 6.57 | 2.90 | 0.39 | 5.86 | 0.48 |
| | poly(I:C)+CpG | 191.12 | 34.79 | 129.16 | 44.66 | 70.93 | 13.18 | 11.66 | 2.15 |
| | poly(I:C)+CpG+R848 | 137.69 | 32.08 | 50.92 | 16.33 | 48.24 | 7.55 | 13.08 | 1.80 |
| | R848+poly(I:C) | 144.09 | 31.62 | 45.71 | 12.46 | 41.47 | 6.27 | 11.77 | 2.43 |
| **cDC1** | w/o TLR ligand | 45.18 | 6.94 | 77.98 | 20.94 | 112.25 | 31.62 | 8.78 | 1.15 |
| | poly(I:C) | 173.65 | 25.08 | 190.94 | 53.70 | 288.08 | 110.02 | 7.33 | 1.05 |
| | poly(I:C)+CpG | 194.04 | 16.93 | 260.64 | 74.30 | 202.36 | 50.32 | 9.77 | 1.76 |
| | poly(I:C)+CpG+R848 | 159.86 | 21.14 | 475.94 | 136.21 | 248.82 | 35.63 | 22.83 | 3.30 |
| | R848+poly(I:C) | 136.79 | 19.60 | 459.91 | 139.28 | 269.59 | 77.14 | 14.66 | 1.57 |
| **cDC2** | w/o TLR ligand | 78.54 | 30.99 | 130.53 | 68.14 | 197.50 | 127.47 | 21.15 | 3.09 |
| | poly(I:C) | 166.62 | 50.93 | 462.38 | 250.41 | 213.14 | 84.00 | 11.80 | 1.38 |
| | poly(I:C)+CpG | 134.39 | 23.96 | 231.96 | 91.09 | 158.96 | 55.97 | 17.28 | 2.16 |
| | poly(I:C)+CpG+R848 | 147.75 | 26.32 | 694.45 | 190.50 | 175.67 | 47.08 | 25.97 | 3.63 |
| | R848+poly(I:C) | 150.42 | 30.76 | 896.76 | 212.17 | 161.86 | 39.08 | 13.73 | 1.68 |
